# Effects of a Standardized Music Intervention on Propofol Requirements and Serum Interleukin-6 and Prolactin in Deeply Sedated Mechanically Ventilated Adults: A Prespecified Secondary Analysis of a Randomized Clinical Trial

**DOI:** 10.3390/healthcare14193300

**Published:** 2026-10-03

**Authors:** Carmen Fernández-Álvarez, María Pilar Mosteiro-Díaz, David Zuazua-Rico, Alba Maestro-González

**Affiliations:** 1 General Intensive Care Unit, Hospital Universitario Central de Asturias, 33006 Oviedo, Spain; carmen.zorera@gmail.com; 2 Faculty of Medicine and Health Sciences, University of Oviedo, 33003 Oviedo, Spain; mmosteirod@uniovi.es (M.P.M.-D.); maestroalba@uniovi.es (A.M.-G.); 3 Instituto de Investigación Sanitaria del Principado de Asturias (ISPA), 33006 Oviedo, Spain

**Keywords:** music, critical care, critical care nursing, respiration, artificial, deep sedation, prolactin, interleukin-6

## Abstract

**Highlights:**

**What are the main findings?**

- In participants receiving propofol, a standardized 60-minute music intervention delivered through active noise-cancelling headphones was associated with a greater reduction in propofol requirements than standard care, while mean BIS values remained within the target range for deep sedation (BIS 40–60) with no evidence of differential BIS trajectories between groups. No statistically significant between-group differences were detected in serum interleukin-6 or prolactin concentrations following a single intervention session, and the intervention was well tolerated without hemodynamic, respiratory, or neurological instability.

**What are the implications of the main findings?**

- Music may represent a safe, low-cost, non-pharmacological adjunct for optimizing sedation management in mechanically ventilated critically ill patients; however, sham-controlled trials with blinded, protocolised sedation titration are required before it can be recommended as a sedative-sparing strategy in routine ICU practice.
- Future studies should determine whether repeated or longer music interventions can influence biological stress responses and longer-term clinical outcomes, while confirming the reproducibility and clinical relevance of the observed reduction in propofol requirements.

**Abstract:**

**Background/Objectives**: Prolonged exposure to sedative agents in critically ill patients receiving invasive mechanical ventilation (IMV) is associated with adverse clinical outcomes. Music interventions have emerged as a promising non-pharmacological strategy to improve patient comfort and potentially reduce sedative requirements. This prespecified secondary analysis of a randomized controlled trial originally powered to detect differences in cortisol concentrations evaluated the effects of a standardized music intervention on sedative requirements and selected inflammatory and neuroendocrine biomarkers in deeply sedated mechanically ventilated adults. **Methods**: An open-label randomized controlled trial involving 110 critically ill adults receiving IMV under deep sedation was conducted. Participants were randomly assigned to a 60-min standardized music intervention delivered through active noise-cancelling headphones or to standard care. Changes in sedative requirements, serum interleukin-6 (IL-6) and prolactin (PRL) concentrations, physiological parameters, and safety outcomes were assessed. **Results**: Among participants receiving propofol at the time of the intervention (n = 84), the music intervention group showed a significantly greater reduction in propofol requirements than those receiving standard care (between-group difference 0.079 mg/kg/h, 95% CI 0.008–0.149; *p* = 0.029). Any reduction in propofol requirements was observed in 46.7% of intervention participants compared with 7.7% of controls. BIS values remained within the target range for deep sedation throughout the intervention period, and no clinically relevant changes were observed in physiological or ventilatory parameters. No significant between-group differences were observed in IL-6 or PRL concentrations after adjustment for baseline values (IL-6: *p* = 0.606; PRL: *p* = 0.093). No intervention-related adverse events were reported. **Conclusions**: A standardized music intervention was associated with reduced propofol requirements without evidence of a between-group difference in BIS trajectory during the intervention period and without clinically relevant physiological instability in mechanically ventilated critically ill patients. No significant between-group effects were observed on IL-6 or PRL concentrations during the study period. As a prespecified secondary analysis of a trial originally powered for a different primary outcome, these findings should be interpreted in that context and warrant confirmation in future randomized trials specifically designed to evaluate music-related effects on sedative requirements. Future randomized trials incorporating appropriate attention-control conditions are warranted to further clarify the specific effects attributable to the musical stimulus.

## 1. Introduction

Critically ill patients receiving invasive mechanical ventilation (IMV) frequently require continuous sedation and analgesia to ensure comfort, facilitate patient–ventilator synchrony, and support the delivery of life-sustaining therapies [1]. Accordingly, sedation is a cornerstone of contemporary critical care management. However, prolonged exposure to sedative agents has been associated with adverse outcomes, including delirium, ICU-acquired weakness, prolonged mechanical ventilation, and extended hospitalization [2]. As a result, current practice increasingly focuses on optimizing sedation while minimizing unnecessary drug exposure without compromising comfort or safety [1,2,3].

Beyond the underlying disease, critically ill patients are exposed to multiple environmental and procedural stressors, including excessive noise, artificial lighting, sleep disruption, social isolation, and repeated invasive procedures [4]. These factors may impair recovery and contribute to patient discomfort. Consequently, growing attention has been directed toward complementary approaches that support patient-centered care while reducing reliance on sedative medications [5,6].

Among these approaches, music interventions have emerged as a promising adjunct to standard care. Their use aligns with the principles of the ABCDEF bundle and broader humanization initiatives aimed at improving patient outcomes and experiences while limiting exposure to potentially burdensome interventions [7,8,9]. Music is also recognized within the Nursing Interventions Classification (NIC) [10], highlighting its relevance as an autonomous, low-cost, safe, and readily accessible nursing intervention. In addition to being safe, inexpensive, and readily accessible, music interventions can be easily incorporated into routine clinical care.

Previous studies have shown that music may reduce anxiety, emotional distress, and discomfort in hospitalized and critically ill patients [11,12,13]. In mechanically ventilated populations, music exposure has been associated with greater relaxation, improved psychological well-being, and lower sedative requirements, suggesting a potential role in sedation management [11,14,15,16]. Proposed mechanisms include modulation of autonomic nervous system activity, attenuation of sympathetic activation, and regulation of neural pathways involved in emotional processing and adaptive physiological responses [17,18,19].

Beyond these clinical effects, music interventions may also influence biological pathways relevant to critical illness. Interleukin-6 (IL-6), one of the most extensively studied inflammatory cytokines, has been consistently associated with infection, organ dysfunction, disease severity, and mortality [20]. Similarly, prolactin (PRL) participates in neuroendocrine and immunomodulatory processes and has been linked to endocrine dysregulation, neurological complications, and unfavorable clinical trajectories [21]. Because these biomarkers reflect distinct physiological domains with established prognostic relevance, they may provide additional insight into the biological effects of non-pharmacological interventions.

Despite growing interest in music-based interventions, evidence regarding their influence on inflammatory and neuroendocrine biomarkers remains limited and inconsistent [12,14,17,22]. Moreover, most available studies have focused on awake or lightly sedated patients, and evidence regarding deeply sedated mechanically ventilated adults remains scarce [11,23]. Consequently, it remains uncertain whether the potential benefits of music on sedation management are accompanied by measurable changes in biological pathways associated with critical illness.

Addressing these uncertainties is particularly relevant from a critical-care nursing perspective, as interventions that reduce sedative exposure without compromising physiological stability may contribute to safer and more humanized care. Therefore, the aim of this randomized clinical trial was to evaluate the effect of a standardized music intervention on sedative requirements and inflammatory and neuroendocrine biomarkers in critically ill adults receiving invasive mechanical ventilation.

## 2. Materials and Methods

### 2.1. Study Design

This study was conducted as an open-label randomized controlled trial with blinded laboratory outcome assessment in the adult intensive care unit of a tertiary-level public university hospital in Spain. The unit comprises 44 beds distributed across general critical care and neurotrauma areas and provides care for patients with a broad range of complex medical and surgical conditions, including severe trauma, multi-organ failure, high-risk postoperative states, transplantation, and critical infections. Participant recruitment and data collection were carried out between October 2023 and June 2025.

The present manuscript reports a prespecified secondary analysis of a randomized clinical trial evaluating the effects of a standardized music intervention in mechanically ventilated critically ill patients (ClinicalTrials.gov: NCT06120660). The trial was registered at ClinicalTrials.gov prior to participant enrolment, and all outcomes reported in the present manuscript were prospectively registered. The first participant was enrolled on 14 October 2023 following ethics committee approval obtained on 10 March 2023.

The parent trial was designed to investigate the effects of the intervention on hormonal and inflammatory responses. Cortisol constituted the primary biomarker outcome on which the original sample size calculation was based, and those results have been reported separately [24]. In contrast, the present manuscript focuses on prospectively registered secondary analyses evaluating sedative requirements together with IL-6 and prolactin concentrations. Although derived from the same randomized cohort, the objectives, analyses, and interpretation presented herein are distinct from those of the cortisol-focused report.

Randomization was performed before participant recruitment using a computer-generated allocation sequence with a 1:1 ratio. A balanced randomization schedule was generated in advance for the planned sample size of 110 participants, resulting in 55 allocations to each study group. No stratification factors were used. An independent researcher not involved in participant recruitment, intervention delivery, data collection, or outcome assessment prepared sequentially numbered sealed envelopes containing group assignments according to the randomization schedule. Allocation concealment was maintained until enrolment, when the investigator responsible for recruitment opened the corresponding envelope to determine group assignment.

Because the intervention involved the use of visible headphones, blinding of bedside healthcare professionals and the researcher responsible for intervention delivery was not feasible. Although all patients remained deeply sedated throughout the intervention period (bispectral index [BIS] 40–60), they were not considered formally blinded for methodological purposes. To minimize detection bias, all biological samples were processed and analysed by laboratory personnel blinded to treatment allocation.

The study was designed and reported in accordance with the Consolidated Standards of Reporting Trials (CONSORT) statement for randomized controlled trials [25].

### 2.2. Participants

Eligible participants were adults (≥18 years) admitted to the intensive care unit for at least 24 h, receiving invasive mechanical ventilation and continuous intravenous sedation as part of routine ICU care, and with a bispectral index (BIS) between 40 and 60 at study inclusion. All participants had an arterial catheter in place to allow blood sampling and showed no evidence of pain at the time of the intervention, defined as a Critical-Care Pain Observation Tool (CPOT) score of 0 [26]. Patients in both study groups were managed according to standard ICU protocols throughout the intervention period.

A small proportion of participants (6 intervention-group participants and 4 control-group participants) received continuous neuromuscular blockade with cisatracurium during the intervention period. In these cases, CPOT assessments were performed before initiation of neuromuscular blockade in accordance with standard ICU practice, and pain evaluation was complemented by physiological indicators and ongoing analgesic management.

Exclusion criteria included severe neurological injury, defined as a Glasgow Coma Scale score < 5 documented before initiation of deep sedation or at ICU admission prior to sedation, or a Marshall computed tomography classification of IV; major psychiatric disorders; active substance abuse or withdrawal syndrome; clinically significant hearing impairment; cranial lesions preventing safe headphone placement; limitations of life-sustaining treatment; anticipated death within the following 24 h; and high-dose vasoactive support, defined as norepinephrine-equivalent dose > 0.1 µg/kg/min.

### 2.3. Sample Size

A pilot study including 40 patients (20 per group) was conducted before trial initiation to estimate the sample size required for the parent randomized clinical trial. The sample size calculation was based on the primary outcome of the parent trial, namely cortisol concentrations. Based on the effect size estimated from the pilot study, a minimum of 50 participants per group was required to achieve 80% statistical power with a two-sided significance level of 0.05. To account for a potential attrition rate of 9%, the target sample size was increased to 55 participants per group, resulting in a total planned sample of 110 participants. Participants included in the pilot study were not included in the final randomized cohort.

**Figure 1:**
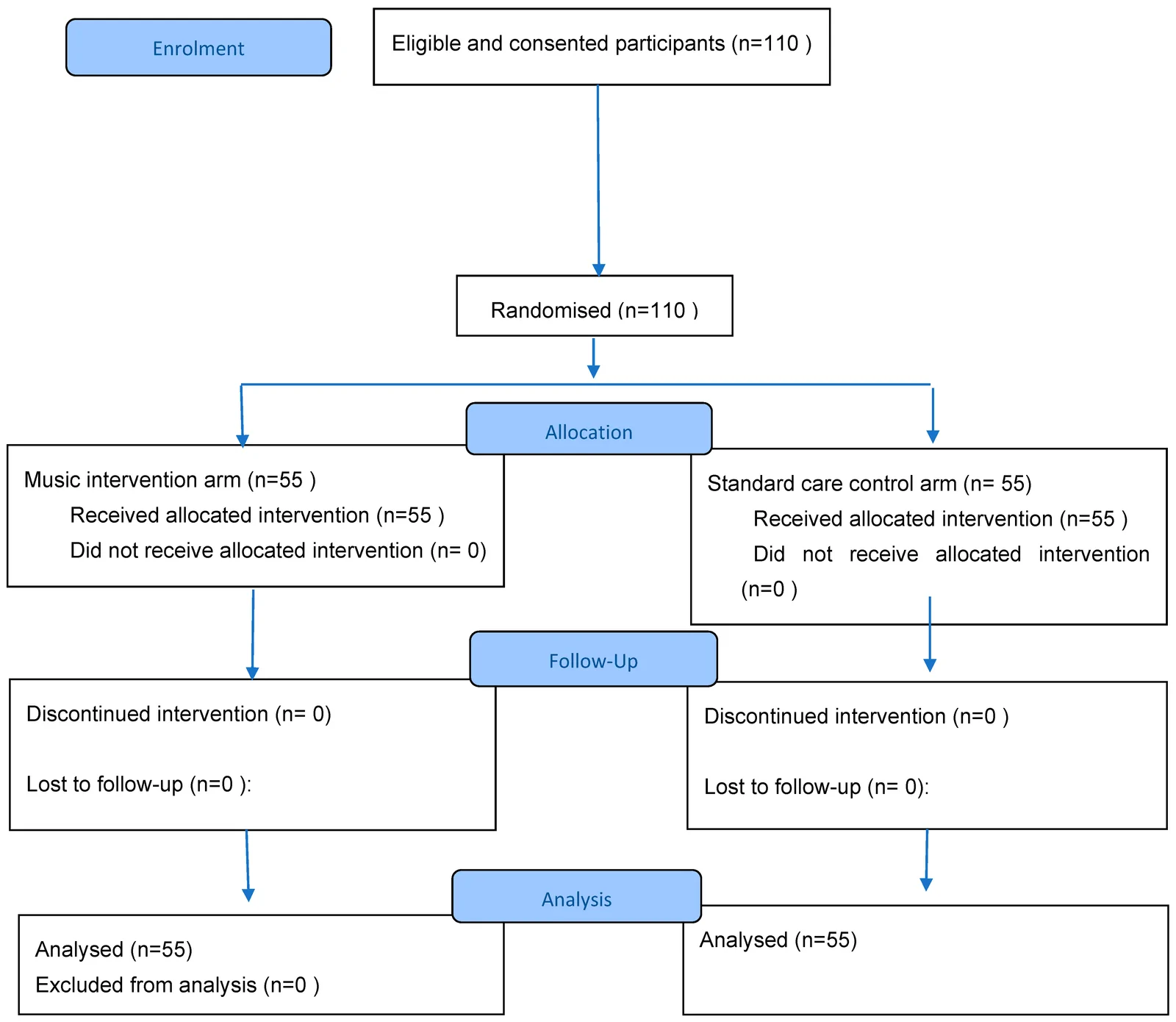
CONSORT 2025 Flow Diagram.

The present study reports a prespecified secondary analysis of sedative requirements together with inflammatory and neuroendocrine biomarkers using the complete trial cohort. Consequently, no specific sample size calculation was performed for the secondary outcomes reported in this manuscript. Participant flow throughout the trial is presented in the CONSORT flow diagram (Figure 1) [25].

### 2.4. Intervention

Participants in the intervention group received a standardized 60-min music intervention delivered through Sony WH-1000XM4 active noise-cancelling headphones (Sony Corporation, Tokyo, Japan). These headphones were selected for their high-fidelity audio reproduction and active noise-cancellation technology, which minimized interference from ambient ICU noise and ensured standardized delivery of the auditory stimulus.

The musical composition, created specifically for the study by a certified music therapist, combined instrumental elements (piano, synthesizer, acoustic guitar, ocean drum, and voice) with natural soundscapes (waves, birdsong, fire crackling, and rain). The piece was characterized by consistently soft dynamics (piano), a slow tempo, and a tonal structure predominantly in C major, selected to promote relaxation and physiological stability. To facilitate reproducibility, the complete musical composition has been made publicly available through Zenodo [27]. Music was delivered at a standardized volume corresponding to 60% of the maximum output of the playback device, a level selected to ensure audibility while avoiding excessive sound exposure.

Participants allocated to the control group remained under standard intensive care conditions without headphones or music, allowing comparison with the usual acoustic environment of the unit.

Environmental noise levels were monitored during intervention sessions. Across all recorded measurements (n = 110), mean environmental noise levels were 45.9 ± 13.9 dB, with maximum noise peaks reaching 92.3 dB, confirming considerable variability in the acoustic environment of the ICU.

The music intervention was administered by the principal investigator, whose role was limited to placing the headphones and initiating the audio playback. The research team was not involved in clinical management or sedation titration during the intervention period.

### 2.5. Sedation Protocol and BIS Monitoring

Sedation depth was standardized using the bispectral index (BIS) (Medtronic, Dublin, Ireland), a processed electroencephalographic parameter commonly used to complement clinical assessment of sedation in critically ill patients. BIS was selected as the study measure for eligibility determination, continuous monitoring, and outcome analysis in order to provide an objective and consistent assessment of sedation depth throughout the intervention period while minimizing patient stimulation during biomarker collection.

BIS values range from 100 (fully awake) to 0 (isoelectric electroencephalogram), with values between 40 and 60 generally considered indicative of deep sedation. BIS provided a standardized electrophysiological measure of sedation depth but was not intended to replace validated clinical sedation scales. Only patients with BIS values within this target range were eligible for inclusion. In both study groups, BIS values were recorded every 10 min throughout the 60-min intervention period.

Although validated clinical sedation scales such as the Richmond Agitation-Sedation Scale (RASS) form part of routine ICU practice, repeated assessment using stimulation-based clinical scales was not performed during the intervention period. Because inflammatory and neuroendocrine biomarkers constituted key study outcomes, minimizing verbal and physical stimulation was considered important to reduce the potential influence of external stimuli on physiological stress responses.

Sedative administration was managed according to the standard ICU sedation protocol prescribed for each patient, with a target BIS range of 40–60. Sedation adjustments were performed by the bedside clinical team, including nursing staff and attending physicians, whenever clinically indicated to maintain the prescribed depth of sedation. The same sedation protocol was applied in both study groups. The research team did not participate in sedation titration decisions and no study-related instructions regarding sedation adjustment were provided to the clinical staff. Because bedside clinicians were aware of treatment allocation, the study design cannot fully distinguish between direct effects of the music intervention and clinician-mediated differences in sedative titration.

### 2.6. Data Collection and Laboratory Analysis

All intervention sessions were conducted between 16:00 and 17:00. A fixed time window was selected to minimize potential circadian influences on biological measurements and to reduce interference with routine nursing activities [28]. Biological samples were obtained immediately before and after the intervention to assess potential acute biomarker responses while minimizing disruption to routine clinical care and avoiding additional blood sampling procedures in critically ill patients through the existing arterial catheter to avoid additional pain or procedural stress.

Samples were processed in the Clinical Biochemistry Laboratory of the participating tertiary-level university hospital by personnel blinded to treatment allocation.

Serum PRL (reported as ng/mL) and IL-6 (reported as pg/mL) concentrations were measured using an electrochemiluminescence immunoassay on a COBAS e801 analyzer (Roche Diagnostics, Mannheim, Germany). All analyses were performed according to the laboratory’s internal quality-control procedures.

### 2.7. Outcome Variables

The outcomes evaluated in this prespecified secondary analysis included medication requirements during the intervention period together with serum interleukin-6 (IL-6; pg/mL) and prolactin (PRL; ng/mL) concentrations.

Medication requirements were assessed as continuous infusion doses of sedative, analgesic, and neuromuscular-blocking agents administered during the study period, including propofol, midazolam, dexmedetomidine, fentanyl, remifentanil, cisatracurium, and inhaled sevoflurane when prescribed.

Infusion rates were originally extracted from bedside infusion pumps and recorded as mL/h. Because medication preparation was standardized according to institutional ICU protocols, infusion rates were converted into pharmacological units using predefined drug concentrations and, when appropriate, the participant’s recorded body weight. Propofol and midazolam infusion rates were expressed in mg/kg/h, fentanyl and remifentanil in µg/kg/h, and dexmedetomidine in µg/h.

For each medication, dose changes were calculated as the difference between pre-intervention and post-intervention infusion rates (pre-intervention dose minus post-intervention dose), such that positive values indicated a reduction in medication requirements during the intervention period. Only infusion rates recorded immediately before the intervention (minute 0) and immediately after completion of the intervention (minute 60) were included in these analyses.

Medication-specific analyses were restricted to participants receiving the corresponding drug at the time of the intervention. Consequently, these analyses should be interpreted as medication-specific exposure analyses within the randomized cohort rather than intention-to-treat comparisons across all randomized participants.

Inflammatory and neuroendocrine outcomes were assessed as changes in serum IL-6 and PRL concentrations measured immediately before and immediately after the intervention period.

Additional variables included BIS values, physiological and ventilatory parameters (heart rate, respiratory rate, oxygen saturation [SpO_2_], systolic blood pressure, diastolic blood pressure, and intracranial pressure [ICP]), together with demographic and clinical characteristics including age, sex, admission diagnosis, duration of mechanical ventilation, ventilatory mode, and Acute Physiology and Chronic Health Evaluation II (APACHE II) score [29].

For analytical purposes, admission diagnoses were grouped into four predefined categories: neurological, respiratory, trauma, and other. Physiological variables and BIS values were recorded every 10 min throughout the 60-min intervention period.

Safety outcomes included the occurrence of intervention-related hemodynamic, respiratory, or neurological instability during the study period. Intervention-related adverse events were defined as any clinically relevant hemodynamic, respiratory, or neurological instability occurring during the intervention period and documented by the attending clinical team.

All outcomes reported in the present manuscript were prospectively registered in ClinicalTrials.gov (NCT06120660).

### 2.8. Ethical Considerations

The study protocol was approved by the Research Ethics Committee of the Principality of Asturias (approval code 2022.537) and registered at ClinicalTrials.gov (NCT06120660). Written informed consent was obtained from the legal representatives of all participants prior to enrolment. The study was conducted in accordance with the Declaration of Helsinki and applicable Spanish data protection regulations.

### 2.9. Statistical Analysis

Descriptive statistics were used to summarize all study variables. Continuous variables were reported as means and standard deviations (SDs) or medians and interquartile ranges (IQRs), depending on data distribution. Categorical variables were summarized as frequencies and percentages.

Baseline characteristics were compared between study groups using Student’s *t*-test, Welch’s *t*-test, or the Wilcoxon rank-sum test for continuous variables, according to data distribution and variance assumptions. Categorical variables were compared using the chi-square (χ^2^) test or Fisher’s exact test, as appropriate.

For analyses of sedative requirements, dose changes were calculated as the difference between pre-intervention and post-intervention infusion rates (pre-intervention dose minus post-intervention dose), with positive values indicating dose reductions during the intervention period. Analyses of individual medications were restricted to participants receiving the corresponding agent at the time of the intervention. Propofol comparisons were performed using Welch’s *t*-test. Midazolam comparisons were performed using the Mann–Whitney U test because dose-change distributions were highly skewed and included a substantial proportion of values close to zero.

Biomarker analyses were performed using analysis of covariance (ANCOVA), with post-intervention concentrations as the dependent variable, study group as the fixed factor, and baseline biomarker values as covariates. Estimated between-group differences and associated *p*-values are reported.

Longitudinal BIS measurements obtained during the intervention period were analyzed using linear mixed-effects models including study group, time (10-min intervals), and the group-by-time interaction as fixed effects, with participant included as a random effect. Participant was included as a random intercept, and time was modelled as a continuous variable representing 10-min intervals across the intervention period.

All available outcome data were analyzed. Medication-specific analyses were restricted to participants receiving the corresponding medication at the time of the intervention and should therefore be interpreted as drug-specific exposure analyses rather than strict intention-to-treat comparisons. Because this manuscript reports a prespecified secondary analysis and multiple outcomes were evaluated, no formal adjustment for multiple comparisons was performed. Results should therefore be interpreted as exploratory and hypothesis-generating.

Statistical significance was established at *p* < 0.05. All analyses were performed using R software (version 4.4.3; R Foundation for Statistical Computing, Vienna, Austria) [30].

## 3. Results

### 3.1. Baseline Characteristics

A total of 110 critically ill patients were included in the analysis, with 55 participants allocated to the control group and 55 to the music intervention group. Baseline demographic, clinical, ventilatory, and biomarker characteristics are summarized in Table 1.

The study population was characterized by advanced critical illness, with a mean age of 63.7 ± 11.7 years and a mean APACHE II score of 21.0 ± 7.0. The mean duration of invasive mechanical ventilation before study inclusion was 4.5 ± 3.5 days, and volume-controlled ventilation was the predominant ventilatory mode (76.4%).

Given the objectives of the present analysis, baseline concentrations of inflammatory and neuroendocrine biomarkers were also examined. Mean IL-6 concentrations were 110.6 ± 105.2 pg/mL in the control group and 148.8 ± 229.2 pg/mL in the intervention group, while mean prolactin concentrations were 45.7 ± 60.3 ng/mL and 46.2 ± 72.7 ng/mL, respectively. Baseline demographic and clinical characteristics were generally similar between study groups. However, IL-6 concentrations showed greater variability in the intervention group, and subsequent biomarker analyses were therefore adjusted for baseline values using ANCOVA.

### 3.2. Sedative Requirements

At the time of the intervention, 39 of 55 participants (70.9%) in the control group and 45 of 55 participants (81.8%) in the intervention group were receiving propofol and were therefore included in the medication-specific propofol analysis.

**Figure 2:**
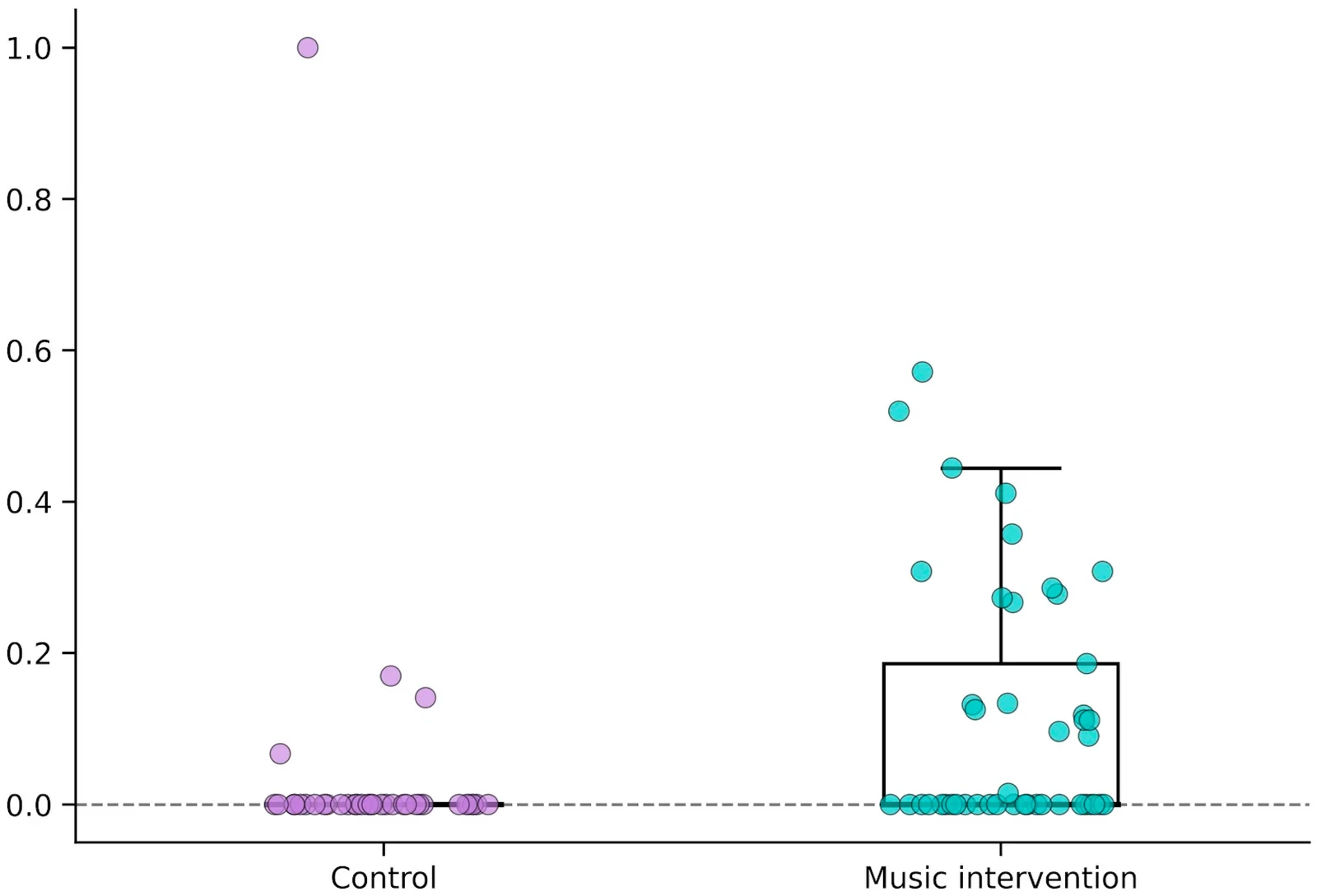
Individual changes in propofol requirements (mg/kg/h) according to study group. Each point represents one participant. Boxplots represent the median and interquartile range. Positive values indicate reductions in propofol requirements during the intervention period.

Among participants receiving propofol at the time of the intervention, the music intervention group showed a significantly greater reduction in propofol requirements than those receiving standard care (0.114 vs. 0.035 mg/kg/h), corresponding to an absolute between-group difference of 0.079 mg/kg/h (95% CI 0.008–0.149 mg/kg/h; *p* = 0.029) (Table 2, Figure 2).

Given the skewed distribution and the large proportion of zero values, a non-parametric comparison was also performed. The distribution of individual propofol dose changes is shown in Figure 2. The reduction in propofol requirements remained significantly greater in the intervention group than in the control group (Mann–Whitney U test, U = 556, *p* < 0.001). Median (IQR) propofol dose reductions were 0.00 (0.00–0.00) mg/kg/h in the control group and 0.00 (0.00–0.186) mg/kg/h in the intervention group (Table 2). As an additional clinically interpretable analysis, any reduction in propofol requirements during the intervention period was observed in 21 of 45 participants (46.7%) in the intervention group compared with 3 of 39 participants (7.7%) in the control group, corresponding to an absolute risk difference of 39.0% (95% CI 22.1% to 55.9%) (Table 2).

Among patients receiving propofol, baseline infusion rates were comparable between groups (Table 3). To account for potential baseline differences, an ANCOVA was performed using post-intervention propofol infusion rates as the dependent variable and baseline propofol infusion rates as a covariate. After adjustment for baseline values, post-intervention propofol requirements remained significantly lower in the intervention group (adjusted mean difference −0.082 mg/kg/h, 95% CI −0.137 to −0.027; *p* = 0.004).

No significant between-group differences were observed for midazolam dose changes (Mann–Whitney U test, W = 195, *p* = 0.296) (Table 2). In addition, no dose changes were observed for dexmedetomidine or fentanyl in either study group during the intervention period. These findings indicate that the reduction in propofol use was not accompanied by a generalized decrease in sedative or analgesic administration.

### 3.3. Inflammatory and Neuroendocrine Biomarkers

Baseline opioid infusion rates (Table 3) and IL-6 and PRL concentrations (Table 4) were broadly comparable between study groups. All randomized participants (55 per group; n = 110) contributed prolactin and IL-6 measurements. In addition, fentanyl requirements remained stable throughout the intervention period, reducing the likelihood that changes in opioid exposure contributed to differences in biomarker concentrations.

In the intervention group, median (IQR) IL-6 concentrations changed from 66.0 pg/mL (30.5–165.0) to 71.0 pg/mL (34.0–189.0), whereas median concentrations in the control group changed from 72.0 pg/mL (35.0–155.0) to 69.0 pg/mL (36.0–163.0). Median (IQR) PRL concentrations increased slightly from 26.2 ng/mL (20.4–48.6) to 27.2 ng/mL (20.1–46.0) in the intervention group and from 22.4 ng/mL (15.1–42.5) to 23.0 ng/mL (14.9–41.1) in the control group.

ANCOVA analyses adjusting for baseline biomarker concentrations showed no significant group effect for IL-6 (adjusted mean difference 1.33 pg/mL; 95% CI −3.76 to 6.42; *p* = 0.606) or PRL concentrations (adjusted mean difference −1.60 ng/mL; 95% CI −3.48 to 0.27; *p* = 0.093).

Consistent with these findings, non-parametric between-group comparisons of biomarker changes showed no significant differences in IL-6 changes (Mann–Whitney U = 1462.0, *p* = 0.765) or PRL changes (Mann-Whitney U = 1198.5, *p* = 0.061).

### 3.4. Physiological Parameters and Safety

Physiological and ventilatory variables were evaluated as safety outcomes to verify that the intervention did not compromise physiological stability. No clinically relevant changes were observed in physiological or ventilatory parameters throughout the intervention period. Systolic and diastolic blood pressure, heart rate, respiratory rate, oxygen saturation (SpO_2_), and intracranial pressure remained stable in both groups, and no intervention-related adverse events were reported.

To evaluate changes in sedation depth during the intervention period, BIS values were analyzed using linear mixed-effects models. No significant group-by-time interaction was observed (β = −0.027, 95% CI −0.068 to 0.015; *p* = 0.210), and no significant effect of time was detected (*p* = 0.598). Mean BIS values remained within the predefined target range for deep sedation (40–60) throughout the intervention period in both groups, and no evidence of differential BIS trajectories between groups was observed. No participant left the predefined BIS target range during the 60-min intervention period, and no randomized participants were excluded from the analyses because of BIS values recorded during the intervention period.

## 4. Discussion

In this randomized clinical trial, a standardized music intervention was associated with reduced propofol requirements without evidence of a between-group difference in BIS trajectory in mechanically ventilated critically ill patients. This effect was reflected not only in a greater mean reduction in propofol requirements but also in a substantially higher proportion of participants experiencing any propofol dose reduction during the intervention period. These findings suggest that music may represent a useful adjunctive strategy associated with lower propofol requirements without evidence of a between-group difference in BIS trajectory during the intervention period.

The sedative-sparing effect identified in the present study is consistent with previous evidence indicating that music interventions may reduce sedative requirements and improve patient comfort in mechanically ventilated adults [11,16,31]. Similar findings were reported by Conrad et al. [32], who observed reductions in sedative requirements following a music intervention in critically ill mechanically ventilated patients. However, most available studies have focused on awake or lightly sedated patients. Our findings extend the current evidence base by suggesting that similar benefits may also be achieved in deeply sedated critically ill patients, a population that remains underrepresented in the literature [22,23].

Importantly, the observed effect was reflected not only in a greater mean reduction in propofol requirements but also in a substantially higher proportion of participants experiencing any propofol dose reduction during the intervention period. Nevertheless, the clinical relevance of these findings should be interpreted cautiously, as the study was not designed to evaluate cumulative sedative exposure or downstream clinical outcomes. Therefore, these results should not be extrapolated to outcomes such as delirium, ICU-acquired weakness, duration of mechanical ventilation, or healthcare utilization, which were not evaluated in the present study [8,33].

Several mechanisms may explain the observed association between the music intervention and reduced propofol requirements. Auditory processing is not completely abolished during sedation, and auditory evoked potentials have been shown to remain responsive to changes in sedative state and anesthetic depth [19]. Consequently, music exposure may modulate autonomic nervous system activity, attenuate sympathetic activation, and reduce stress-related responses [15,17]. However, because sedation titration was performed by unblinded bedside clinicians according to routine clinical practice, the present study was not designed to distinguish between direct physiological effects of music and clinician-mediated changes in sedative titration. Even in the absence of conscious awareness, these mechanisms may contribute to lower pharmacological requirements to maintain a comparable level of sedation.

Active noise-cancelling headphones were intentionally used to standardize auditory exposure and minimize the potentially confounding influence of ambient ICU noise. Environmental noise monitoring performed during intervention sessions demonstrated substantial variability in the ICU acoustic environment, with peak noise levels exceeding 90 dB. This approach ensured that participants in the intervention group were exposed to a consistent auditory stimulus and reduced variability attributable to environmental noise. However, because a headphone-only control group was not included, the relative contribution of music exposure and acoustic isolation cannot be completely disentangled. This limitation is particularly relevant given the substantial variability observed in the ICU acoustic environment, with peak noise levels exceeding 90 dB. Therefore, the observed reduction in propofol requirements may reflect the effects of music exposure, acoustic isolation, or a combination of both. Future studies should incorporate sham-headphone control conditions to distinguish the contribution of these components, ideally using either a three-arm design (music plus headphones, headphones alone, and standard care) or identical headphones with and without music.

In contrast to the reduction in sedative requirements, no significant between-group differences were detected in acute serum IL-6 or PRL responses during the study period. These findings should be interpreted within the context of a single 60-min intervention and a sampling strategy designed to capture immediate biomarker responses rather than sustained biological modulation. Furthermore, the selected sampling schedule may not have been optimal for detecting delayed or transient biological responses. Although both biomarkers were selected because of their prognostic relevance and their involvement in inflammatory and neuroendocrine pathways [21,34,35,36], their biological behaviour may limit the detection of short-term intervention-related effects. Compared with rapidly responding stress mediators such as cortisol, IL-6 and PRL exhibit substantial interindividual variability and may require longer observation periods to demonstrate measurable changes [37].

The interpretation of prolactin findings should be made with caution. Opioid administration is known to stimulate prolactin secretion, and although baseline fentanyl and remifentanil infusion rates were comparable between groups and no relevant changes in opioid requirements were observed during the intervention period, residual confounding cannot be completely excluded.

Previous studies evaluating the effects of music on inflammatory and neuroendocrine biomarkers have produced heterogeneous findings. While reductions in IL-6 concentrations have been reported in some settings [17,32,38], findings regarding neuroendocrine biomarkers have been less consistent.

Notably, Beaulieu-Boire et al. [14] reported significant reductions in cortisol and prolactin concentrations following a music intervention in mechanically ventilated ICU patients. This study is particularly relevant because it evaluated a similar critically ill population, included inflammatory and neuroendocrine biomarkers, and incorporated a sham-MP3 control condition. In contrast, no significant between-group effects on prolactin concentrations were observed in the present study.

Several methodological differences may help explain these discrepant findings. Beaulieu-Boire et al. [14] used a randomized crossover design with repeated music exposure and a sham-MP3 control condition in mechanically ventilated sedated patients. In addition to reporting reductions in cortisol and prolactin concentrations, the authors observed a non-significant trend toward lower opioid requirements. In addition, the substantial biological variability of prolactin and its pulsatile secretion pattern may have reduced our ability to detect short-term intervention-related effects.

Other investigations have reported minimal or non-significant biomarker effects, particularly in critically ill populations [39]. The absence of detectable biomarker changes in the present study may also reflect the complex physiological environment of critical illness. Critically ill patients are exposed to multiple pharmacological and non-pharmacological factors that may influence inflammatory and neuroendocrine pathways, potentially obscuring subtle biological responses to music interventions. Furthermore, previous reviews have highlighted the inconsistency of biomarker findings in this field, with effects often varying according to patient characteristics, clinical context, and biomarker selection [40].

The intervention demonstrated a favourable safety profile. No clinically relevant changes were observed in physiological or ventilatory parameters, and no intervention-related adverse events occurred. These findings are consistent with previous studies reporting good tolerability of music interventions in mechanically ventilated and critically ill patients [15,41]. The absence of hemodynamic, respiratory, or neurological instability further supports the feasibility of incorporating music into routine intensive care practice.

Beyond its physiological effects, the incorporation of music into intensive care practice supports broader efforts to humanize critical care [7,8,9,42]. As a low-cost, non-pharmacological, and easily implemented intervention, music may represent a promising strategy for promoting patient-centred care in critically ill patients. Given its inclusion within the Nursing Interventions Classification [10], music may be readily integrated into nurse-led care protocols, reinforcing the central role of critical care nurses in delivering holistic and evidence-based care.

Several limitations should be acknowledged. First, the intervention consisted of a single 60-min session and biomarker measurements were limited to the pre-intervention and immediate post-intervention periods. Consequently, delayed, cumulative, or transient biological effects may have gone undetected, particularly for biomarkers characterized by substantial interindividual variability and complex temporal kinetics, such as IL-6 and prolactin.

Second, the study was conducted as an open-label trial because blinding of bedside clinicians was not feasible. Sedation management was performed as part of routine clinical care according to the therapeutic goals established for each patient and was not determined by the study protocol. Although identical BIS-guided sedation procedures and target ranges were applied in both groups, clinicians responsible for sedation management were aware of treatment allocation. In addition, detailed records of individual titration events, including their timing, magnitude, and the specific BIS values prompting dose adjustments, were not prospectively collected. Therefore, the possibility of performance bias cannot be completely excluded.

Third, this study represents a prespecified secondary analysis of a randomized controlled trial originally powered to detect differences in cortisol concentrations. The sample size was not specifically calculated for the sedative, inflammatory, or neuroendocrine outcomes evaluated here, and no formal adjustment for multiple comparisons was performed. Consequently, these findings should be considered exploratory. In particular, analyses involving IL-6 and prolactin concentrations may have been underpowered to detect small-to-moderate effects, and the absence of significant differences should be interpreted as inconclusive rather than evidence of absence of an effect.

Finally, the heterogeneous nature of the study population may limit the generalizability of the findings. Although BIS provided a standardized and objective measure of sedation depth, concurrent assessment using a validated clinical sedation scale such as the Richmond Agitation-Sedation Scale (RASS) was not performed during the intervention period. This decision was made because repeated RASS assessments would have required verbal and/or physical stimulation of deeply sedated patients during biomarker collection. Such stimulation could itself influence physiological stress responses and potentially affect neuroendocrine and inflammatory biomarker concentrations. Nevertheless, the absence of concomitant clinical sedation assessment may limit direct comparison with studies combining electrophysiological and clinical measures of sedation.

## 5. Conclusions

In participants receiving propofol at the time of the intervention, a standardized music intervention was associated with reduced propofol requirements and with a substantially greater likelihood of any propofol dose reduction during the intervention period, without evidence of a between-group difference in BIS trajectory and without clinically relevant physiological instability. No significant between-group effects were observed for IL-6 or prolactin concentrations following a single 60-min intervention.

Although these findings derive from a prespecified secondary analysis and require confirmation in future sham-controlled trials, they suggest that music may represent a promising non-pharmacological adjunct for optimizing sedation management in critically ill mechanically ventilated adults. Further research should determine whether repeated interventions can reduce cumulative sedative exposure and improve clinically relevant outcomes.

## Figures and Tables

**Table 1:** Baseline demographic and clinical characteristics of the study population.

Characteristic	Control Group (n = 55)	Music Intervention Group (n = 55)
Age (years), mean (SD)	65.05 (10.38)	62.33 (12.92)
Sex, n (%)		
Male	36 (65.5)	38 (69.1)
Female	19 (34.5)	17 (30.9)
Days of invasive mechanical ventilation prior to intervention, mean (SD)	4.80 (3.44)	4.18 (3.55)
APACHE II score, mean (SD)	21.25 (7.65)	20.74 (6.29)
Primary diagnosis, n (%)		
Neurological	25 (45.5)	20 (36.4)
Respiratory	14 (25.5)	15 (27.3)
Traumatic	9 (16.4)	10 (18.2)
Other	7 (12.7)	10 (18.2)
Ventilatory mode, n (%)		
Volume-controlled ventilation	41 (74.5)	43 (78.2)
Pressure support ventilation	14 (25.5)	11 (20.0)
Pressure-controlled ventilation	0 (0.0)	1 (1.8)
Baseline biomarkers		
Interleukin-6 (pg/mL), mean (SD)	110.60 (105.21)	148.76 (229.16)
Prolactin (ng/mL), mean (SD)	45.74 (60.31)	46.18 (72.71)

Abbreviations: APACHE II, Acute Physiology and Chronic Health Evaluation II; SD, standard deviation.

**Table 2:** Changes in sedative requirements according to medication-specific exposure groups ^1,2^.

Sedative	Group	n	Mean Change (SD)	Median (IQR)	Between-Group Difference (95% CI)	*p*-Value ^3^
Propofol ^4^	Control	39	0.035 (0.163)	0.00 (0.00–0.00)		
	Intervention	45	0.114 (0.161)	0.00 (0.00–0.186)	0.079 (0.008–0.149)	0.029
Midazolam	Control	17	0.020 (0.037)	0.011 (0.000–0.020)		
	Intervention	19	0.002 (0.032)	0.004 (−0.015–0.022)	Not estimable	0.296
Dexmedetomidine	Control	4	0.000 (0.00)	0.00 (0.00–0.00)		
	Intervention	4	0.000 (0.00)	0.00 (0.00–0.00)	Not estimable	Not estimable

^1^ Abbreviations: SD, standard deviation; CI, confidence interval. ^2^ Medication-specific analyses included only patients receiving the corresponding drug at the time of the intervention. Some participants received more than one sedative agent; therefore, denominators are not independent and should not be summed across rows. ^3^ Propofol comparisons were performed using Welch’s *t*-test. Midazolam comparisons were performed using the Mann–Whitney U test because dose-change distributions were highly skewed and included a substantial proportion of values close to zero. ^4^ A non-parametric sensitivity analysis confirmed a significantly greater reduction in propofol requirements in the intervention group than in the control group (Mann–Whitney U = 556, *p* < 0.001). No dose changes were observed for dexmedetomidine in either group; therefore, between-group differences could not be estimated.

**Table 3:** Baseline sedative and analgesic infusion rates among participants receiving each medication.

Sedative	n (Control)	n (Music)	Control Group, Mean (SD)	Music Intervention Group, Mean (SD)	*p*-Value
Propofol (mg/kg/h)	39	45	0.876 (0.605)	0.859 (0.694)	0.901
Midazolam (mg/kg/h)	17	19	0.115 (0.079)	0.108 (0.055)	0.746
Fentanyl (µg/kg/h)	49	50	1.034 (0.418)	0.987 (0.402)	0.564
Dexmedetomidine (µg/h)	4	4	68.0 (38.1)	190.4 (145.9)	0.177
Remifentanil (µg/kg/h)	3	2	6.045 (3.426)	4.667 (1.886)	0.604

Abbreviations: SD, standard deviation. Infusion rates correspond to pre-intervention values. Because only participants receiving the corresponding medication at the time of the intervention were included in each analysis, sample sizes differed across medications. Denominators should not be summed across rows. Results for dexmedetomidine and remifentanil should be interpreted with caution due to the limited number of exposed patients. No dose changes were observed for cisatracurium, remifentanil, fentanyl, or sevoflurane during the intervention period; consequently, these agents were not included in the analysis of dose changes.

**Table 4:** Between-group comparison of post-intervention biomarker concentrations adjusted for baseline values (ANCOVA).

Biomarker	Adjusted Mean Difference	95% CI	*p*-Value
IL-6 (pg/mL)	1.33	−3.76 to 6.42	0.606
PRL (ng/mL)	−1.60	−3.48 to 0.27	0.093

Abbreviations: CI, confidence interval. Non-parametric analyses of biomarker changes confirmed the absence of significant between-group differences for IL-6 (Mann–Whitney U = 1462.0, *p* = 0.765) and PRL (Mann–Whitney U = 1198.5, *p* = 0.061).

## Data Availability

The dataset generated and analyzed during the current study has been deposited in Zenodo (https://doi.org/10.5281/zenodo.20407074). Access to the dataset is restricted to protect participant confidentiality. Qualified researchers may request access through the repository. Requests will be considered on a case-by-case basis and access will be granted when the proposed use is scientifically justified and consistent with the ethical approvals and data protection requirements governing the study.

## Abbreviations

The following abbreviations are used in this manuscript:

APACHE II	Acute Physiology and Chronic Health Evaluation II
BIS	Bispectral Index
CI	Confidence Interval
CONSORT	Consolidated Standards of Reporting Trials
CPOT	Critical-Care Pain Observation Tool
ICU	Intensive Care Unit
IL-6	Interleukin-6
IMV	Invasive Mechanical Ventilation
NIC	Nursing Interventions Classification
PRL	Prolactin
SD	Standard Deviation
SpO_2_	Peripheral Oxygen Saturation
